# Borderline personality disorder diagnosis in a new key

**DOI:** 10.1186/s40479-019-0116-1

**Published:** 2019-12-02

**Authors:** Abby L. Mulay, Mark H. Waugh, J. Parks Fillauer, Donna S. Bender, Anthony Bram, Nicole M. Cain, Eve Caligor, Miriam K. Forbes, Laurel B. Goodrich, Jan H. Kamphuis, Jared W. Keeley, Robert F. Krueger, John E. Kurtz, Peter Jacobsson, Katie C. Lewis, Gina M. P. Rossi, Jeremy M. Ridenour, Michael Roche, Martin Sellbom, Carla Sharp, Andrew E. Skodol

**Affiliations:** 1 Medical University of South Carolina, 29C Leinbach Drive, Charleston, SC 29407 USA; 2 Oak Ridge National Laboratory (ORNL) & University of Tennessee Knoxville, Knoxville, TN 37996 USA; 3 University of Tennessee Knoxville, Knoxville, TN 37996 USA; 4 Tulane University, 6823 St. Charles Ave., Bldg. 92, New Orleans, LA 70118 USA; 5 Lexington, USA; 6 Rutgers University, Graduate School of Applied and Professional Psychology, 152 Frelinghuysen Rd, Piscataway, NJ 08854-8020 USA; 7 Columbia University, 1501 Riverside Drive, New York, NY 10032 USA; 8 Macquarie University, Balaclava Rd., Macquarie Park, NSW 2109 Australia; 9 Knoxville, USA; 10 University of Amsterdam (UvA), Nieuwe Achtergracht, 129B, 1001 NK Amsterdam, Netherlands; 11 Virginia Commonwealth University, 806 West Franklin Street, Box 842018, Richmond, VA 23284-2018 USA; 12 University of Minnesota, 101 Pleasant St SE, Minneapolis, MN 55455 USA; 13 Villanova University, 800 Lancaster Avenue, Villanova, PA 19085 USA; 14 Institute of Neuroscience and Physiology, University of Gothenburg, Gothenburg, Sweden; 15 Austen Riggs Center, 25 Main Street, P.O. Box 962, Stockbridge, MA 01262 USA; 16 Department of Psychology, Personality and Psychopathology Research group Vrije Universiteit Brussel (VUB), Brussels, Belgium; 17 Penn State Altoona, 3000 Ivyside Park, Altoona, PA 16601 USA; 18 University of Otago, PO Box 56, Dunedin, 9054 New Zealand; 19 University of Houston, 3695 Cullen Boulevard Room 126, Houston, TX 77204-5022 USA; 20 University of Arizona, 1501 N. Campbell Avenue, PO Box 245017, Tucson, AZ 85724 USA

**Keywords:** Borderline personality disorder, Alternative model for personality disorders, Personality assessment, DSM-5, Personality disorder

## Abstract

**Background:**

Conceptualizations of personality disorders (PD) are increasingly moving towards dimensional approaches. The definition and assessment of borderline personality disorder (BPD) in regard to changes in nosology are of great importance to theory and practice as well as consumers. We studied empirical connections between the traditional DSM-5 diagnostic criteria for BPD and Criteria A and B of the Alternative Model for Personality Disorders (AMPD).

**Method:**

Raters of varied professional backgrounds possessing substantial knowledge of PDs (*N* = 20) characterized BPD criteria with the four domains of the Level of Personality Functioning Scale (LPFS) and 25 pathological personality trait facets. Mean AMPD values of each BPD criterion were used to support a nosological cross-walk of the individual BPD criteria and study various combinations of BPD criteria in their AMPD translation. The grand mean AMPD profile generated from the experts was compared to published BPD prototypes that used AMPD trait ratings and the DSM-5-III hybrid categorical-dimensional algorithm for BPD. Divergent comparisons with DSM-5-III algorithms for other PDs and other published PD prototypes were also examined.

**Results:**

Inter-rater reliability analyses showed generally robust agreement. The AMPD profile for BPD criteria rated by individual BPD criteria was not isomorphic with whole-person ratings of BPD, although they were highly correlated. Various AMPD profiles for BPD were generated from theoretically relevant but differing configurations of BPD criteria. These AMPD profiles were highly correlated and showed meaningful divergence from non-BPD DSM-5-III algorithms and other PD prototypes.

**Conclusions:**

Results show that traditional DSM BPD diagnosis reflects a common core of PD severity, largely composed of LPFS and the pathological traits of anxiousness, depressively, emotional lability, and impulsivity. Results confirm the traditional DSM criterion-based BPD diagnosis can be reliably cross-walked with the full AMPD scheme, and both approaches share substantial construct overlap. This relative equivalence suggests the vast clinical and research literatures associated with BPD may be brought forward with DSM-5-III diagnosis of BPD.

## Background

Psychodiagnosis serves many masters. The clinician, for example, wants a system that is practical for work in practical settings (e.g., hospitals, outpatient practices and clinics, agencies, forensics, etc.); in other words, a diagnostic system that has clinical utility, one that values matters of communication, ease of use, and treatment planning [1]. On the other hand, the researcher often privileges scientific concerns (which nonetheless also may be studied with idiographic methods), like reproducibility and the statistical relationships between measurements of the phenomena of interest (i.e., construct validity [1]). Because these stakeholders tend to value and emphasize different elements and even models of diagnostic systems, conceptualizations of psychiatric disorders reflect tensions in the field. Regarding personality disorders (PD), stakeholder tensions have been described as dialectics [2]. For the specific diagnosis of BPD, perhaps because of the historical and ongoing clinical importance of the syndrome, these tensions seem particularly acute [3]. Concerns and debates about how to formulate the diagnosis of BPD often pivot on the current interest in dimensionalizing diagnostic systems.

The contemporary movement toward dimensionalization of diagnosis in psychopathology [4] and in PD [5] represents a paradigm shift in the field away from the traditional categorical and syndrome approach [6]. It is widely understood that the diagnosis of BPD is of vast clinical, scientific, and public health importance. Given this, it is not surprising that significant flashpoints have emerged over the merits of dimensionalizing BPD. Proponents of different approaches to PD often invoke the issue of differential clinical utility or scientific validity in support of points of view [5, 7].

Several dimensional approaches to PD diagnosis exist [8–12]; however, the Alternative Model for Personality Disorders (AMPD) in Section III (i.e., the Emerging Measures and Models section) of the *Diagnostic and Statistical Manual of Mental Disorders* (Fifth Edition [DSM-5]) [13] represents a dimensional approach that is receiving significant attention [14]. Nonetheless, meaningful concerns have been levied against the AMPD and other dimensional approaches on a variety of grounds [3, 7, 15–18].

### The current study

Recognizing that dimensional and traditional categorical-syndrome formulations of BPD diagnosis accent certain features and under-emphasize others, we were interested in studying the correspondence between how the two approaches are construed by clinicians and psychopathologists well acquainted with PD and the AMPD. For BPD, this can be stated as, “How do knowledgeable PD experts translate traditionally defined BPD diagnostic criteria with the scheme of the AMPD?” To study this, we invited individuals with expertise in personality, psychopathology, and PD assessment and treatment to characterize the DSM-5 Section II (traditional categorical) BPD criteria with the elements of the DSM-5 Section III AMPD, permitting a cross-walking between the two models. The Level of Personality Functioning Scale (LPFS) of AMPD Criterion A and the 25 pathological personality traits of AMPD Criterion B were mapped on to the nine individual diagnostic criteria of DSM-5 Section II BPD.

These data then permitted detailed examination of the relative contributions of Criterion A and Criterion B in the representation of the nine BPD diagnostic criteria. They also enabled study of how different combinations of BPD criteria (that met the threshold for the diagnosis of BPD) are represented in the metric of the AMPD. This included study of the DSM BPD criteria with reference to published base rates of occurrence and clinician views of the importance of different diagnostic criteria. The nine BPD criteria, translated into the AMPD metric and aggregated, were also examined for correspondence with published AMPD whole-person prototype ratings for BPD, to other AMPD representations of BPD, and to AMPD hybrid categorical-dimensional diagnostic algorithms. Thus, we were able to reckon our AMPD BPD criteria ratings with different and important lines of research within the vast literature on diagnostic modeling and criteria compositions for BPD. Our analyses involved both convergent and divergent (non-BPD PD) correlational comparisons. A factor analysis of the BPD criteria in the AMPD metric was performed and compared with results from published factor analytic studies of the traditional BPD criteria. Collectively, our different methodological comparisons serve to connect our approach with several of the many ways BPD and BPD diagnosis have been studied in the past.

To the extent the elements of the Section II and Section III models can be shown to substantially interdigitate, it follows that the empirical findings and clinical lore associated with the nomological nets of categorical and dimensional diagnostic conceptions of BPD may then be transposable. This may also help to clarify potential tradeoffs between clinical utility and scientific validity of these two diagnostic paradigms. The current study also extends existing literature in several respects. First, the current study utilized expert ratings of BPD, as opposed to self-report methods. Second, we focus on individual DSM-5 Section II BPD criteria, rather than whole-person or “prototypical” patient ratings. Finally, the current study examined Criterion A (i.e., level of personality functioning) and Criterion B (25 pathological trait facets) of the AMPD. As has been noted, the burgeoning literature on the AMPD often reflects studies of the AMPD traits and self-report methods [19]. Our detailed crosswalk between the DSM-5 Section II BPD criteria with both Criterion A and B of the AMPD thus extends findings such as those of Evans and Simms [20] and Waters et al. [21], which focused on trait ratings and self-report methods.

## Method

### Participants

An international team (*N* = 20) consisting of 16 clinical psychologists, one advanced doctoral student in clinical psychology, one clinical psychology researcher, and two psychiatrists, formed the rater pool. Rather than attempting to select a representative sample of all mental health professionals, raters were recruited so as to provide a wide range of years of clinical experience, theoretical orientations, international status, and professional work settings, as well as clear expertise in personality theory and assessment. Raters included two members of the DSM-5 Personality and Personality Disorders Work Group and a consultant to the International Classification of Diseases (ICD) 11th Edition (ICD-11) PD Committee, prominent researchers in PD and psychopathology, and practicing professionals with extensive clinical experience in diagnosing and treating PD. Very importantly, the panel of raters included advocates of dimensional diagnostic schemes as well as those who value traditional conceptions. All evaluators were knowledgeable and experienced with the AMPD.

The average years of clinical experience was about 20 years. Theoretical orientations ranged, but the percentage of self-identified orientations, averaged across all participants, were as follows: psychodynamic (43%), cognitive-behavioral therapy (26%), interpersonal (11%), and other orientations (< 4%). Raters collectively self-identified as spending 34.5% of their professional time in clinical work and 64.5% time in research. Eight of the raters reported the majority of their professional activity was clinical service (ranging from 60 to 100%). All participants were asked to what extent they felt the AMPD effectively captured the syndrome of BPD using a 0–5 scale. The mean rating was 4.1 (*SD* = .64), indicating generally favorable views of the AMPD approach. In addition, an outside expert (also a member of the DSM-5 Personality and Personality Disorders Work Group) who did not participate in the rating procedure was asked to provide an expert opinion “back translation” of the raters’ AMPD depiction of BPD.

### Measure

The AMPD was deconstructed into the four domains of the LPFS (i.e., identity [ID], self-direction [SD], empathy [EM], and intimacy [IN]) of Criterion A and the 25 pathological trait facets of Criterion B. Participants were tasked with characterizing each diagnostic criterion of the nine BPD criteria with the four domains of Criterion A and the 25 pathological trait facets of Criterion B. Because we wished to examine interrelations between traditional DSM BPD diagnosis and the AMPD scheme with as much granularity as practical, we devoted significant attention to the four domains of the LPFS, rather than focusing solely on the LPFS as a unitary index of PD. As the LPFS uses a 0 to 4 rating in the DSM-5, this metric was maintained for the task. Thus, raters were asked to use the following metric when rating each of the BPD criteria according to Criterion A: 0 = *lack of representation of the BPD criterion within the LPFS*; 1 = *limited presence of the BPD criterion within the LPFS*; 2 = *moderate presence of the BPD criterion within the LPFS*; 3 = *significant presence of the BPD criterion within the LPFS*; 4 = *very significant presence of the BPD criterion within the LPFS*. Raters were also asked to evaluate the BPD criteria with the 25 pathological trait facets of Criterion B. To be consistent with previous literature [22], we used the following scale: 0 = *lack of representation of the BPD criterion within the trait*; 1 = *limited presence of the BPD criterion within the trait*; 2 = *moderate presence of the BPD criterion within the trait*; 3 = *significant presence of the BPD criterion within the trait*.

### Procedure

Participants were contacted by electronic mail and invited to participate in a study of clinician ratings of BPD. Twenty (84%) of the 24 potential raters who were contacted agreed to participate and completed all tasks. In part, we believe this high participation rate reflects the expertise of the panel of raters, their interest in the aims of the project, and its importance to the field at large. Raters were sent a spreadsheet in which each DSM-5, Section II BPD criterion was reproduced verbatim, and they were asked to evaluate each criterion with the elements of the AMPD, referring to the DSM-5 Section III text definitions of Criterion A and Criterion B. For the task, the raters were instructed to consider an abstract target person or patient who demonstrated (1) all general PD criteria (DSM-5, p. 663) *and* (2) PD as defined by the AMPD inclusion criterion of a moderate (i.e., rating of 2) or greater rating on the LPFS in two out of four domains. This step was taken to help raters situate consideration of the BPD criteria within a PD-relevant clinical context, rather than potentially referencing a general population distribution of PD-related dimensions or variables.

Once these data were returned to the three lead authors, initial means for the four LPFS domains and the 25 pathological trait facets for each of the BPD criteria were calculated. Next, these summary data were emailed back to the participants and, following a modified Delphi design format [23, 24], they were invited to consider making any revisions to the summary means they felt were indicated, based on feedback from the group data. Thus, each rater both provided their ratings independently and they were later able to suggest changes in AMPD group means, if they felt it was indicated. This latter step afforded an opportunity for the group to iterate to a final, collective AMPD group mean.

In order to provide a “back-translation” from the final grand mean AMPD BPD profile (averaged across all nine BPD criteria), a blinded, outside expert^1^ in the AMPD (i.e., not involved in the rating procedure), who was also a member of the DSM-5 PD Work Group, was given the mean AMPD profile and asked to describe the personality, PD characteristics, and any DSM-IV/5 PD diagnoses suggested by the AMPD profile.

### Statistical analyses

We studied inter-connections between traditional BPD diagnostic criteria (and diagnosis) and the element so the AMPD in multiple ways. The first step was to establish the descriptive statistics for the rater pool. To evaluate rater agreement, intraclass correlation coefficients (ICCs) for the AMPD ratings of the nine BPD criteria were calculated. As satisfactory levels of rater agreement were achieved (see below), mean AMPD values for each of the BPD criteria were computed and subjected to further analysis. The mean LPFS ratings for the BPD criteria were examined in relation to rater variables (e.g., years of clinical experience, work setting, theoretical orientation). The inter-correlations, including a summary principal component analysis (PCA), between the nine BPD criteria (defined by AMPD ratings) were examined. Next, our grand mean AMPD profile for BPD was also correlated with “AMPD trait profiles” drawn from other empirical studies of BPD. These included (1) the mean of 10 PD experts^2^ who were asked to characterize a prototypical BPD patient with the 25 traits of the AMPD from Waugh, Bishop, and Schmidt [25]; (2) results from Anderson, Sellbom, and Shealey’s [26] study of 105 mental health clinicians who rated a “typical” BPD patient with the AMPD traits; (3) Morey, Benson, and Skodol’s [27] study of 337 clinicians, which offered AMPD and DSM-IV criterion count sum correlations for various DSM PD syndromes; and (4) the DSM-5-III hybrid categorical-dimensional algorithm for BPD (defined as positive for anxiousness, depressivity, emotional lability, hostility, impulsivity, risk taking, separation insecurity with a rating of 3 [0–3 scale] and all other traits set at 0). These data also permitted divergent comparisons of our grand mean AMPD profile for BPD with other (non-BPD) DSM-5-III PD algorithms and with respect to other published non-BPD AMPD profiles.

As the DSM-IV is a polythetic nosology, numerous combinations of criteria can yield the diagnosis of DSM-IV BPD.^3^ We studied this multiplicity by computing the AMPD BPD profiles our data yielded when BPD was defined by various configurations of BPD criteria. These configurations were defined by: (1) the reported base rate (BR) occurrence of BPD criteria from Grilo and colleagues [28]; (2) clinician-rated causal centrality of BPD criteria of Kim and Ahn [29]; and (3) the rank ordered LPFS severity of the BPD criteria, as found in the present study. For the BR criteria comparisons, individual AMPD-rated BPD criteria were compiled as a function of five, seven, eight, and nine BPD criteria (the latter is the grand mean).^4^ The different combinations of AMPD-defined BPD criteria were then compared by Pearson product-moment correlations and ICCs.

## Results

### Rater agreement and the grand mean AMPD profile for the BPD criteria

The inter-rater agreement for the raters’ evaluation of each of the nine BPD criteria with the elements of Criteria A and B was quantified by ICCs (2-way, random effects, consistency, average measures). Because the interests of the current study were generally in the mean values of raters’ evaluations of BPD criteria, and because group-level and correlational analyses including (idiographic) profile analyses were employed, we used the consistency ICC to benchmark rater agreement (unless otherwise noted). Table 1 shows ICCs for Criterion A (the LPFS) and Criterion B (the 25 trait pathological trait facets) for each BPD criterion. Regarding the LPFS, eight of the nine DSM-5 BPD criteria demonstrated strong levels of agreement, except for BPD criterion 8 (i.e., intense anger), which was not as strong. For the 25 traits of Criterion B of the AMPD, all ICCs were strong. The global mean ICC across the four domains of the LPFS and all nine BPD criteria was strong, as it was for the 25 traits. This robust level of rater agreement supported combining clinician ratings and computing mean AMPD metrics for each of the nine BPD criteria across the 20 raters. In turn, a grand mean across all nine BPD criteria was also found (see Table 2).^5^

**Table 1:** Initial rater ICCs

BPD Criteria	LPFS ICCs	Trait ICCs
Criterion 1 (avoidance of abandonment)	.94	.96
Criterion 2 (unstable relationships)	.94	.93
Criterion 3 (identity disturbance)	.94	.88
Criterion 4 (impulsivity)	.91	.97
Criterion 5 (recurrent suicidal behavior)	.81	.97
Criterion 6 (affective instability)	.92	.98
Criterion 7 (chronic emptiness)	.95	.97
Criterion 8 (intense anger)	.46	.97
Criterion 9 (paranoia/dissociative symptoms)	.87	.96

*N* = 20 raters. *ICC* = mean consistency intraclass correlation coefficient, *BPD =* borderline personality disorder

**Table 2:** Mean ratings across BPD criteria for Criterion A (Level of Personality Functioning) and Criterion B (traits)

	Mean Rating and Standard Deviation Time 1	Mean Rating and Standard Deviation Time 2
Level of Personality Functioning Domain		
Identity	2.73 (1.09)	2.72 (.63)
Self-Direction	2.36 (1.16)	2.37 (.55)
Empathy	2.16 (1.25)	2.18 (.52)
Intimacy	2.27 (1.26)	2.24 (.71)
Pathological Trait-Facet		
Anhedonia	1.07 (1.18)	1.06 (.84)
Anxiousness	**1.61 (1.16)**	**1.59 (.64)**
Attention-Seeking	1.01 (1.01)	1.02 (.63)
Callousness	.75 (.86)	.78 (.54)
Cognitive and Perceptual Distortion	.96 (1.06)	.96 (.77)
Deceitfulness	.31 (.55)	.32 (.18)
Depressivity	**1.51 (1.21)**	**1.51 (.95)**
Distractibility	.72 (.92)	.73 (.39)
Eccentricity	.39 (.69)	.38 (.39)
Emotional Lability	**2.05 (1.10)**	**2.08 (.67)**
Grandiosity	.57 (.82)	.57 (.37)
Hostility	1.32 (1.16)*	1.32* (.77)
Impulsivity	**1.70 (1.13)**	**1.73 (.89)**
Intimacy Avoidance	.58 (.84)	.59 (.26)
Irresponsibility	.71 (.96)	.72 (.70)
Manipulativeness	.79 (.94)	.80 (.67)
Perseveration	.57 (.96)	.57 (.33)
Restricted Affectivity	.33 (.72)	.32 (.48)
Rigid Perfectionism	.26 (.61)	.28 (.14)
Risk Taking	1.14* (1.17)	1.17* (.93)
Separation Insecurity	1.13* (1.10)	1.14* (.71)
Submissiveness	.47 (.74)	.48 (.41)
Suspiciousness	1.08 (1.17)	1.06 (.87)
Unusual Beliefs and Experiences	.41 (.78)	.42 (.64)
Withdrawal	.61 (.87)	.61 (.43)

*N* = 20 raters. Bolded entries are mean ratings that round to “2” on a 0–3 scale (= > 1.5). Italicized and starred entries are traits in the AMPD BPD algorithm that did NOT reach this level in the current study. The ICC between time one and time two was 1.0, indicating no significant changes in ratings

As previously noted, we implemented a partial Delphi methodology [23, 24] by providing the group means to participants for review and potential revision. Modifications made by participants were updated in the dataset, and group means were re-calculated with the Delphi-adjusted data. Most participants (*n* = 14) did not modify ratings, and the differences between the raters’ initial and the final Delphi-adjusted ratings (averaged over BPD criteria) were effectively nil (see Table 2; absolute agreement ICC between Time 1 and Time 2 = 1.0).

Overall rater means and standard deviations (*SD*) were then calculated for the four domains of the LPFS (see Table 2). A previous study of LPFS reliability used a LPFS value of > 1.5 to approximate the AMPD threshold criterion of LPFS of 2 [31, 32], and we also utilized this value in our analyses. Each of the nine individual BPD criteria achieved the LPFS threshold value of (rounded) 2 (*M* = 2.35; *SD* = .35; range 1.86–3.01). Table 3 shows the breakdown of mean LPFS and the LPFS domain values by the individual BPD criteria, and Table 4 shows the breakdown of mean pathological trait facet values by the individual BPD criteria. However, a total of six raters provided ratings with LPFS values of < 2 (based on the mean of identity, self-direction, empathy, intimacy calculated across all nine BPD criteria). The BPD criteria LPFS values for three raters were between 1.61 and 1.97, which round to the whole number 2, the threshold for PD in the AMPD. Collectively, the average of these 6 (“low value”) raters’ mean LPFS values was 1.51. In view of this result and in the interest of maximizing input from all expert participants, subsequent calculations used data from all 20 raters.

**Table 3:** Mean LPFS values for BPD criteria

BPD Criterion	ID	SD	EM	IN	LPFS Sum
Criterion 1 (avoidance of abandonment)	2.35	1.75	2.60	3.25	2.49
Criterion 2 (unstable relationships)	2.15	1.60	2.75	3.20	2.43
Criterion 3 (identity disturbance)	3.55	2.85	1.90	1.65	2.49
Criterion 4 (impulsivity)	1.80	2.65	1.60	1.40	1.86
Criterion 5 (recurrent suicidal behavior)	2.70	2.75	2.15	2.00	2.40
Criterion 6 (affective instability)	2.72	1.80	1.55	1.55	1.91
Criterion 7 (chronic emptiness)	3.15	2.75	1.65	1.85	2.08
Criterion 8 (intense anger)	2.40	2.30	2.65	2.55	2.48
Criterion 9 (paranoia/dissociative symptoms)	3.65	2.90	2.75	2.75	3.01
Mean	2.72	2.37	2.18	2.24	

*BPD* = borderline personality disorder, *ID =* identity, *SD =* self-direction, *EM =* empathy, *IN =* intimacy, *LPFS =* Level of Personality Functioning Scale

**Table 4:** Mean trait facet values for BPD criteria

	C1	C2	C3	C4	C5	C6	C7	C8	C9
Anh	.50	.50	.85	.60	2.05	1.10	2.85	.35	.75
Anx	2.4	1.40	1.20	1.20	1.55	2.75	1.05	.85	1.95
AS	1.95	1.30	.65	1.50	1.80	.70	.30	.70	.30
Call	.70	1.35	.35	1.10	.90	.30	.30	1.75	.25
Cog	.80	.50	1.30	.65	1.10	.45	.50	.50	2.85
Dect	.60	.60	.35	.30	.30	.20	.20	.35	.10
Dep	1.35	.95	1.30	.70	2.95	2.45	2.70	.35	.80
Dist	.35	.25	.80	1.45	.50	.90	.60	.55	1.15
Eccen	.30	.25	.40	.25	.30	.25	.15	.15	1.14
EL	2.55	2.45	1.60	1.65	2.45	3.00	.85	2.50	1.65
Gran	.60	1.35	.80	.45	.30	.35	.10	.75	.40
Hos	1.15	1.80	.65	.90	1.1	1.75	.35	2.95	1.25
Impul	1.50	1.55	1.1	2.95	2.70	2.0	.35	2.50	.90
IA	.35	.65	.45	.25	.70	.35	.95	.65	.95
Irres	.20	.40	.35	2.35	.90	.55	.25	1.25	.25
Man	1.90	1.40	.20	.85	1.55	.40	.15	.65	.10
Per	1.25	.45	.10	.80	.80	.40	.45	.45	.45
RA	.05	.05	.20	.25	.20	.05	1.55	.05	.50
RP	.30	.50	.45	.30	.25	.05	.30	.10	.25
RT	1.20	.55	.65	2.90	2.35	.45	.40	1.65	.40
SI	2.85	1.45	1.05	.60	1.1	1.15	.90	.55	.60
Sub	1.45	.70	.60	.30	.30	.30	.40	.10	.20
Sus	1.75	1.60	.45	.20	.30	.65	.40	1.40	2.75
Unu	.25	.25	.40	.15	.20	.10	.20	.10	2.10
WD	.35	.40	.45	.25	.80	.30	1.40	.30	1.20
Mean	1.07	.91	.67	.92	1.10	.84	.70	.86	.95

*ANH* = Anhedonia, *Anx* = Anxiety, *AS* = Attention-Seeking, *Call* = Callousness, *Cog =* Cognitive and Perceptual Distortion, *Dect* = Deceitfulness, *Dep* = Depressivity, *Dist* = Distractibility, *Eccen =* Eccentricity, *EL* = Emotional Lability, *Gran* = Grandiosity, *Hos =* Hostility, *Impul =* Impulsivity, *IA =* Intimacy Avoidance, *Irres =* Irresponsibility, *Man* = Manipulativeness, *Per* = Perseveration, *RA* = Restricted Affectivity, *RP* = Rigid Perfectionism, *RT* = Risk Taking, *SI* = Separation Insecurity, *Sub* = Submissiveness, *Sus* = Suspiciousness, *Unu* = Unusual Beliefs and Experiences, *WD* = Withdrawal, *C1* = Criterion 1, *C2* = Criterion 2, *C3* = Criterion 3, *C4* = Criterion 4, *C5* = Criterion 5, *C6* = Criterion 6, *C7* = Criterion 7, *C8* = Criterion 8, *C9* = Criterion 9

Table 5 shows the BPD diagnostic criteria associations with the four domains of the LPFS. Although each LPFS domain (averaged across the BPD criteria) was “positive for PD” with a rounded mean > 2, the LPFS domain of identity rounded to “3,” whereas self-direction, empathy, and intimacy round to “2.”

**Table 5:** Correlations between AMPD-defined BPD criteria

BPD Criteria	One	Two	Three	Four	Five	Six	Seven	Eight	Nine
Criterion 1 (avoidance of abandonment)	1								
Criterion 2 (unstable relationships)	.85	1							
Criterion 3 (identity disturbance)	0.58	0.61	1						
Criterion 4 (impulsivity)	0.35	0.36	0.48	1					
Criterion 5 (recurrent suicidal behavior)	0.59	0.56	0.70	0.71	1				
Criterion 6 (affective instability)	0.66	0.65	0.73	0.48	0.77	1			
Criterion 7 (chronic emptiness)	0.29	0.29	0.71	0.17	0.72	0.55	1		
Criterion 8 (intense anger)	0.57	0.81	0.59	0.67	0.61	0.62	0.23	1	
Criterion 9 (paranoia/dissociative symptoms)	0.43	0.51	0.74	0.17	0.34	0.49	0.49	.48	1

*BPD* = borderline personality disorder. *r* = .43 significant at .05; *r* = .48 significant at .01

The above analyses of LPFS ratings for the BPD criteria were calculated by averaging across raters. Alternatively, LPFS ratings can also be studied by tallying individual raters’ frequency of positive-rated LPFS values (two or more of the four LPFS domains positive) for five or more BPD criteria (the BPD diagnostic threshold). This comparison showed that 19 of the 20 raters (95%) rated the LPFS positive for five or more BPD criteria. In terms of the individual BPD criteria considered positive on the LPFS (two or more for two or more LPFS domains), the results showed the following percentages and numbers of raters: 75% and 15 raters (criterion 6), 80% and 16 raters (criterion 4), 85% and 17 raters (criterion 5 and 8), 90% and 18 raters (criterion 3 and 9), and 95% and 19 raters (criterion 1, 2, and 7). Similarly, percentages ranged from 75% and 15 raters (criterion 3 and 5) to 90% and 18 raters (criterion 9) for participants who viewed a BPD criterion reflecting a positive value (ratings of two or more) on all four LPFS domains.

Next, overall rater means and *SD*s were calculated for the 25 pathological trait facets of Criterion B (see Table 2). If a trait’s mean score was 1.50 or above, it was considered significant because it rounded to 2, a common practice for determining relevance to PD with the AMPD (e.g., [33]). These mean ratings showed that AMPD-defined BPD was characterized by the four Criterion B traits of anxiousness, depressivity, emotional lability, and impulsivity. In this regard, it should be noted that the hybrid categorical-dimensional algorithm for BPD in the AMPD additionally includes the traits of hostility, risk taking, and separation insecurity; these traits did not achieve our cut off threshold value of 1.50.

Rater characteristics were also explored in relation to severity ratings given to AMPD depictions of the BPD criteria. We examined associations with variables of academic vs. clinical practice work setting, therapeutic orientation, years of clinical experience, and raters’ opinions of the quality of the AMPD system. Among these several variables, only the psychodynamic orientation showed a significant correlation with severity of AMPD judgments (.49 [*p* < .03] for the full AMPD; .47 for traits [*p* < .04]; .43 for LPFS [*p* < .06]). No other participant variable showed a significant association with severity indices. However, for the six raters whose LPFS domain means were below the exact PD threshold of 2.0 (range .97 to 1.97, *M* = 1.51 [*SD* = .39]), their work setting was 97% academic (3% practice) and self-identified theoretical orientation was 23% psychodynamic. In contrast, the other raters (*n* = 14) self-identified as 52% academic (48% practice) work setting and 52% psychodynamic theoretical orientation, and they had a mean LPFS rating of 2.75 (*SD* = .34). The means of both groups of raters were significantly different (*t* [18] = 7.17, *p* < .001).

Given the findings of strong rater agreement for characterizing BPD criteria with the elements of the AMPD, these data also provide a way to study differential patterning of AMPD variables with respect to different combinations of the BPD criteria. These analyses begin with study of the intercorrelation of the BPD criteria when characterized by the AMPD model.

### Principal components analysis

In order to summarize the intercorrelations between our AMPD-defined BPD criteria, a PCA was conducted. This PCA with oblimin rotation of the nine BPD criteria was computed on the universe of the means of the 29 elements of the AMPD. An oblique rotation was selected because the BPD criteria reflect correlated features of the syndrome. This procedure in effect constitutes a Q-factor analysis for profile agreement [34]. The AMPD ratings of the BPD criteria variables exhibited no problematic skew distributions (all skew values < 2), the Kaiser-Meyer-Olkin test (.67) was acceptable, and Bartlett’s Test of Sphericity (sig. < .001); thus, all indicated it was permissible to perform a PCA on these data. Inspection of scree plots and the criterion of eigenvalue greater than 1 both indicated a two-component solution was parsimonious and reasonable in this exploratory analysis, accounting for 73% of the variance.^6^ See Table 6 for the component values. The first component was large and accounted for 60% of the variance, and, notably, seven of the nine BPD criteria loaded above .48, with the exception of BPD criteria 7 and 9. The resulting two PCA components were correlated (.46) and demonstrated meaningful patterns of loadings. Component 1 (C1) was largely defined by BPD criterion 2 (unstable interpersonal relationships), 5 (recurrent self-harm), and 8 (intense anger). Component 2 (C2) was mainly defined by BPD criteria 3 (identity disturbance), 7 (chronic emptiness), and 9 (dissociation/paranoia). These two components were labeled as dyscontrol and acting out (C1) and self-identity disturbance (C2). Nonetheless, the two components were highly correlated, revealing only moderate distinctiveness and some BPD criteria showed large cross loadings on both components (e.g., BPD criteria 3 and 5). The Pearson product-moment correlations for the BPD criteria, defined by AMPD ratings, are presented in Table 5. In sum, the BPD criteria, characterized by the AMPD scheme, demonstrate very substantial intercorrelations.

**Table 6:** PCA of BPD criteria in AMPD Metric

BPD Criteria	Component 1 (Dyscontrol - Acting Out)	Component 2 (Self Identity Disturbance)
Criterion 1 (avoidance of abandonment)	.79	.49
Criterion 2 (unstable relationships)	**.85**	.49
Criterion 3 (identity disturbance)	.68	**.89**
Criterion 4 (impulsivity)	.77	.18
Criterion 5 (recurrent suicidal behavior)	**.80**	.62
Criterion 6 (affective instability)	.77	.70
Criterion 7 (chronic emptiness)	.31	**.89**
Criterion 8 (intense anger)	.**90**	.38
Criterion 9 (paranoia/dissociative symptoms)	.43	**.78**

Bolded items are considered key markers for the given component. An oblimin rotation was used*. PCA* = principal components analysis, *BPD* = borderline personality disorder

Another way to express the psychometric relationships between the AMPD-rated BPD criteria is to correlate each AMPD-BPD criterion with the mean of the other eight AMPD-BPD criteria (minus the index criterion). These item-total correlations for the eight AMPD-BPD criteria were as follows: .69, .76, .85, .53, .80, .81, .53, .74, and 57. Results show that strong internal consistency obtains over the BPD criteria (defined by AMPD ratings), and these findings are congruent with the high degree of shared variance seen in the PCA.

### Criterion-aggregated trait profile and whole-person AMPD prototypes convergence

An individual DSM diagnostic criterion, even when combined with other criteria, may or may not carry the same information as a diagnostic characterization at the whole-person level. In order to study these issues, the AMPD pathological trait grand means computed for the aggregated nine individual BPD criteria (*each of which are specific criteria* and not whole-person BPD targets) from our present study were compared with whole-person clinician ratings of AMPD traits associated with BPD abstracted from two other studies in the literature. These studies provided rating data based on 10 PD experts of Waugh et al. [25] and 105 clinicians of Anderson et al. [26]. Both studies asked the clinician to evaluate a person with prototypical BPD using the 25 AMPD pathological trait facets. Agreement for the 10 expert PD raters in Waugh et al. [25] was strong: the ICC (2-way random effects, consistency agreement) was .59 single and .94 mean. This level of agreement justified taking the group mean as an expert AMPD trait profile of prototypal of BPD. The Anderson et al. [26] study did not permit interrater reliability data to be described similarly.

The Waugh et al. [25], Anderson et al. [26], and current study grand mean AMPD trait profiles were compared for agreement using ICCs. The two-way random effects, absolute agreement mean ICCs for these studies were good to excellent. The current study AMPD trait results for the aggregated BPD criteria correlated with the 10 experts’ prototype ratings of Waugh et al. [21], with an ICC = .63 (good). The current study’s results correlated with the Anderson et al. [22] prototype ratings with an ICC = .75 (excellent). For comparison, we note the 10 experts of Waugh et al. [25] correlated with the 105 clinicians of Anderson et al. [26] at ICC = .83 (excellent). Thus, our BPD AMPD profile created by aggregating trait ratings across the BPD nine criteria, which summarizes the ratings for individual BPD criteria rather than whole-person ratings for BPD, nonetheless shows strong resemblance to the results of the prototype rating method.

### Additional convergent associations

We examined correlations between additional ways of depicting BPD with the AMPD. First, we operationalized the DSM-5 AMPD hybrid categorical-dimensional algorithm for BPD by assigning the designated traits (i.e., anxiousness, depressivity, emotional lability, hostility, impulsivity, risk taking, and separation insecurity) a value of “3,” and “0” for non-included traits. Second, we examined the AMPD trait profile for BPD found in Morey et al.’s [27] study of 337 clinician ratings of patients using the AMPD and DSM-IV PD criteria. For comparison purposes, we also examined associations with an AMPD trait profile devised out of the meta-analytic mean correlation values for each of the 25 AMPD traits associated with BPD diagnosis reported in Waters et al. [21]. The Waters et al. [21] meta-analysis pooled data from studies that used AMPD and consisted of 19 samples and, for the BPD diagnosis, nearly 8000 total subjects. However, it should be emphasized that the meta-analysis mainly reflected data from Personality Inventory for the DSM-5 (PID-5 [35]) self-report methods (e.g., 30 of their 37 correlation matrices were with PID-5 self-report data). Thus, this index of Criterion B traits associated with BPD diagnosis is not fully comparable to the Waugh et al. [25], the Anderson et al. [26], or the Morey et al. [27] indices for BPD because they were based on clinician whole-person ratings as opposed to mainly self-report assessment defined BPD as with Waters et al. [21]. Thus, increased method of measurement variance is expected to affect comparisons with the Waters et al. [21] results.

Table 7 presents our mean AMPD trait profile (aggregated overall nine BPD criteria) correlated with the data from Waugh et al. [25], Anderson et al. [26], the DSM-5-III hybrid algorithm, the Morey et al. [27] data for BPD ratings, and the Waters et al. [21] meta-analytic and mainly PID-5-defined BPD profile. The aggregated BPD criterion AMPD rating profile from the current study was most highly associated with the Waugh et al. [25] expert AMPD prototype, but the differences between the correlations with the Anderson et al. [26], DSM-5 hybrid algorithm, and Morey et al. [27] results are not significantly different. However, the aggregated BPD criterion AMPD rating profile was least associated with the findings from Waters et al. [21] showing a correlation of .62 (significant at *p* < .02, one-tailed, Z = 2.16).

**Table 7:** Pearson correlations between BPD rating studies

Rater Study	1.	2.	3.	4.	5.	6.
1. Waugh et al.	1					
2. Anderson et al.	.81	1				
3. DSM-5 Hybrid Model	.82	.76	1			
4. Morey et al.	.83	.92	.71	1		
5. Waters et al.	.70	.38	.47	.54	1	
6. Current Study	.88	.82	.82	.82	.62	1

All correlations are significant at *p* < .01; *N* = 25 AMPD pathological trait-facets

### Divergent correlations

It is informative to contrast the above convergent correlational results with those for divergent associations with the aggregated BPD criterion AMPD rating profile. This was examined in different ways. First, we compared the aggregated BPD criterion profile with the five other DSM-5-III categorical-dimensional PD algorithms (antisocial, avoidant, narcissistic, obsessive-compulsive, and schizotypal). This was also done by marking a defining trait with a “3” and a non-defining trait with “0” for each PD algorithm. This analysis permits divergent relationships with the AMPD algorithms to be discerned with respect to the BPD algorithm. Secondly, we compared our aggregated BPD criterion AMPD profile with the AMPD trait profile for each of the Morey et al. [27]^7^ and the Waters et al. [21] non-BPD DSM-IV PDs. The mean divergent correlation for our aggregated BPD criteria AMPD profile with the (non-BPD) AMPD hybrid categorical-dimensional PD algorithms was −.19. The comparable mean divergent correlation for the Morey et al. [27] and Waters et al. [21] non-BPD AMPD profiles was −.05. These comparisons show that the aggregated BPD criterion AMPD profile is substantially different from AMPD algorithms for other PDs and with respect to non-BPD AMPD profiles derived from other studies.

### AMPD profiles from various configurations of BPD criteria

**Fig. 1:**
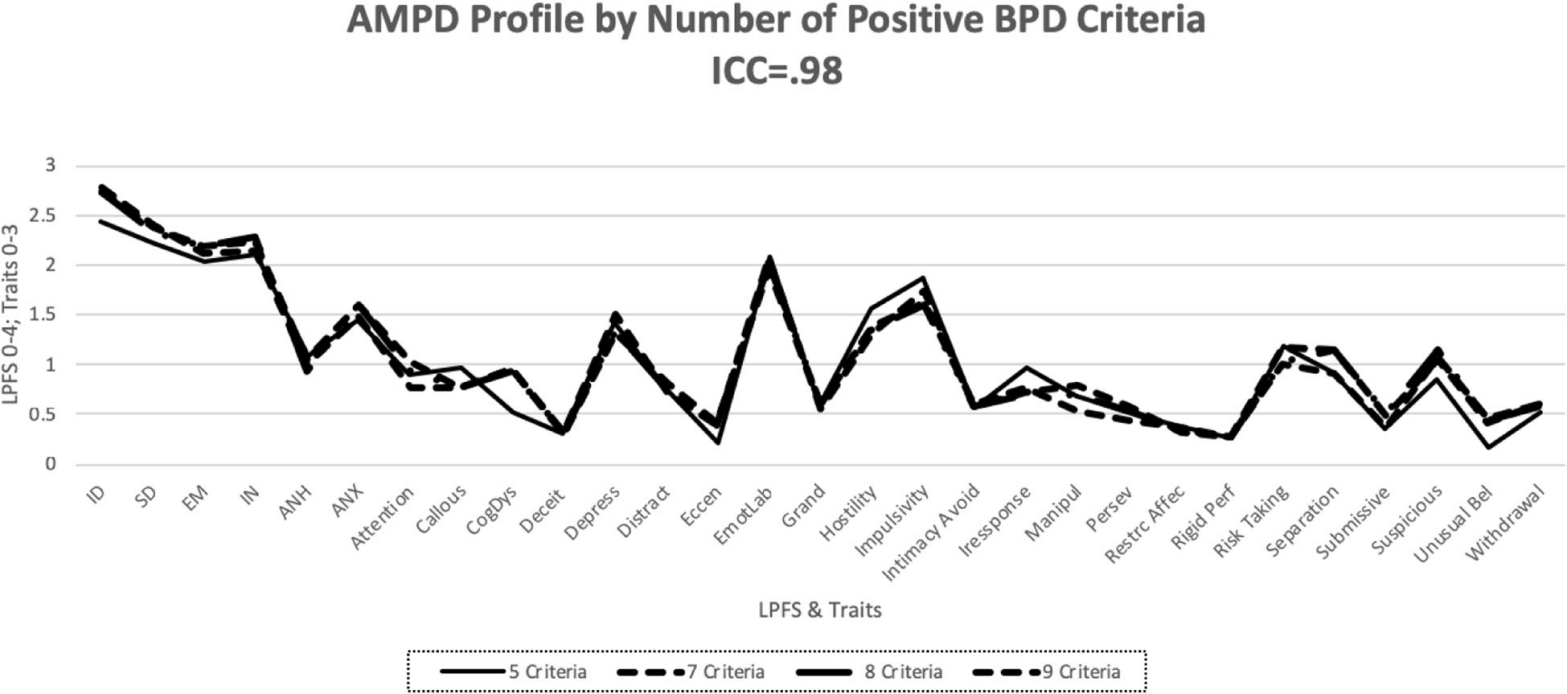
AMPD = Alternative Model for Personality Disorders. BPD = borderline personality disorder. ICC = intraclass correlation coefficient

In view of the fact the polythetic nosology of the DSM-IV BPD criteria can produce 256 different combinations of positive diagnoses, we explored the implications of different combinations of the BPD criteria when they are expressed in the AMPD rating metric. We compiled mean AMPD profiles based on five, seven, eight, and nine BPD criteria, with these numbers of criteria organized by their empirical base rates (BR) of occurrence of the criteria, based on data from Grilo et al. [28]. For example, for the five-criteria BPD AMPD profile, we calculated the average of the mean AMPD ratings for the first five BPD criteria in rank order of frequency of diagnosis. Figure 1 shows the relationship between AMPD profiles of BPD computed for different configurations of criteria. Importantly, all profiles are extremely similar. This can be quantified by the ICC (2-way random effects, absolute agreement) single rater agreement for AMPD BPD profiles based on five, seven, eight, and nine criteria, which produced an ICC of .98. Thus, common BR occurrences of BPD criteria yielding the diagnosis of BPD show very similar AMPD profiles.

The empirical BRs of the BPD criteria in persons diagnosed as BPD are not necessarily the same as the BPD criteria clinicians believe are most central to making a diagnosis of BPD. To explore this, we used data from Kim and Ahn’s [29] study of clinicians’ ratings of the causal centrality and importance of DSM diagnostic criteria. We developed a series of AMPD profiles generated from the rank order of the clinician rated causal centrality of the BPD criteria. This was done for each of five, seven, eight, and nine BPD criteria. These AMPD BPD profile configurations were highly similar and showed strong correspondence with the BR-configured AMPD profiles. For example, the single ICC (2-way, random effects, absolute) between the BR-determined and the rank ordered causally central BPD criteria for five BPD criteria was .95. The ICC between the five causally central criteria and all nine BPD criteria (the grand mean) was .96.^8^

Finally, we compared the AMPD BPD profile associated with the five highest LPFS-rated BPD criteria (BPD criteria 9, 1, 3, 8, 2) with the above ways to configure BPD and their associated AMPD profiles. This “LPFS severity” BPD AMPD profile correlated .90 with the AMPD profile of the first five BR-ordered criteria, and .97 with the first five causally central BPD criteria (Pearson *r*s). The single ICC values were .89 and .96, respectively. As with the previous comparisons of different configurations of BPD criteria, the five most severe BPD criteria determined by LPFS value differed little from BR or causally central (five) criteria determined AMPD profiles.

### Qualitative Back-translation of the grand mean AMPD BPD profile

The outside expert, who was blinded to the details of our study, described the grand mean AMPD profile for BPD found from our clinician ratings in the following manner:

The most elevated facets are depressivity, anxiety, emotional lability, impulsivity. This combination resembles the criteria for DSM-IV borderline PD, particularly in the sense that impulsivity is mixed with emotional lability. LPFS domains are all elevated, particularly identity disturbance. This is also consistent with the DSM-IV^9^ borderline PD criteria.

## Discussion

To our knowledge, this is the first study to examine the nine criteria of BPD (at the level of the individual diagnostic criterion) in relation to both the LPFS and the 25 pathological trait facets of the AMPD. It is also the first AMPD study of BPD to focus on multiple conceptually meaningful combinations of the specific BPD diagnostic criteria (as opposed to whole-person or prototype ratings of BPD) while using the full AMPD scheme and without reliance on self-report instrumentation. Numerous important findings emerged.

First, we found that clinicians and researchers of varying theoretical orientations and professional work settings can characterize the nine criteria of BPD with the AMPD scheme with excellent overall agreement. For the LPFS (Criterion A), eight of the nine BPD criteria evidenced excellent levels of rater agreement, with a single exception (i.e., fair rater agreement for BPD criterion 8, which refers to intense anger). Although only one BPD criterion, cross-walked with the LPFS, showed less than excellent rater agreement, this result nonetheless reminds clinicians of the importance of developing an adequate understanding of the scope and purpose of the LPFS, as well as practice with the measure before rendering clinical PD diagnoses with the AMPD. With respect to the literature on applying the LPFS, studies have reported varying levels of inter-rater reliability [32, 36–42], but they generally reveal fair levels of agreement among raters using the LPFS. However, it should be noted these studies used various forms of the LPFS, types of raters, application targets, and methods of ascertainment. It is known that method factors are highly relevant to the assessment of diagnostic reliability [43]. Hence, it is essential to specify the “what,” “how,” “who,” and “where” of rater reliability with the LPFS (and AMPD).

Most recent research findings indicate clinical application of the LPFS can be performed with acceptable levels of interrater reliability, particularly after training in its use [31, 39]. Interestingly, even though our raters possessed very strong and relevant expertise, six of the 20 raters did not achieve the exact threshold value of 2 for mean LPFS across their ratings of the BPD criteria. But, these findings can be stated in another, meaningful way, with respect to diagnosis of BPD. The number of raters who provided positive LPFS ratings (two or more of the four domains) for five or more of the nine BPD criteria was 19 of the 20 raters. Thus, combining the BPD criteria to yield the diagnosis of BPD, 95% of the raters gave positive LPFS ratings.

The ratings for LPFS at the domain level illustrate an important observation. That is, the mean rating rounded to a whole number for the ID domain of the LPFS was ‘3’ whereas the SD, EM, and IN domains were ‘2.’ This result for the domain of ID highlights the importance of identity dysfunction in DSM BPD diagnosis. This observation is consistent with studies of clinicians’ conceptualization of BPD [29], as well as classic formulations of BPD [44], which influenced modern DSM formulations of the syndrome, and recent emphases on the role of identity in the developmental psychology of BPD [45].

Interestingly, very few rater characteristics were associated with making more or less AMPD severity judgments for the BPD criteria. Although our comparisons do not exhaust the many ways different rater characteristics could be studied in relation to applying the AMPD criteria for BPD, they suggest the AMPD may be relatively agnostic with respect to clinician characteristics, such as years of clinical experience, their opinion on the merits of the AMPD system, and type of professional work setting. This is an important finding, given that some have expressed concerns the LPFS may be too difficult or theoretically complex [37]. We did find that self-identified psychodynamic orientation was moderately and significantly correlated with rendering more severe AMPD ratings of the BPD criteria. This observation recalls the conceptual heritage of the LPFS, which drew heavily from psychodynamic research traditions, as well as social-cognitive developmental theory [46]. Relatedly, Mulay, Cain, Waugh, et al. [47] found that knowledgeable raters considered the elements of Criterion A to reflect greater psychodynamic (and personological) personality paradigms relative to Criterion B, which was seen as more reflective of the multivariate and empirical paradigms. It may be that greater familiarity or comfort with psychodynamic thinking sensitizes clinicians to more differentiated (or at least more severe) LPFS judgments of psychopathology. In this regard, it should be noted that the most conservative raters (with LPFS grand mean ratings < 2) strongly identified with an academic work setting and much less of the psychodynamic orientation.

**Fig. 2:**
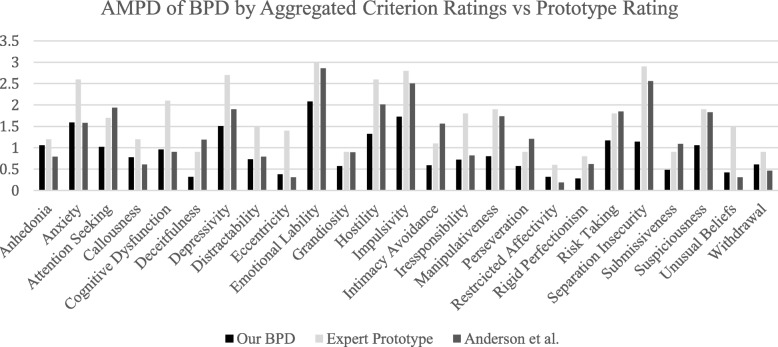
AMPD = Alternative Model for Personality Disorders. BPD = borderline personality disorder

Amongst the most important contributions of the current study is the overall AMPD portrayal of BPD across all nine criteria (see Fig. 2). This AMPD profile of BPD shows that only four of the seven official DSM-5 Criterion B traits were rated as clinically significant (i.e., trait mean score of > 1.5). These were anxiousness, depressivity, emotional lability, and impulsivity. Notably, the other three AMPD-defining traits for BPD (separation insecurity, hostility, and risk taking) were not significant. This finding suggests that clinicians, when evaluating *individual BPD diagnostic criteria*, find anxiousness, depressivity, emotional lability, and impulsivity the key trait markers when the diagnostic criteria are combined to yield threshold BPD. This finding is not identical to that from clinicians’ ratings made at the whole-person or prototype level [25, 26]. When the rating target is a whole person prototype, other traits (such as attention seeking, cognitive and perceptual dysregulation, intimacy avoidance, irresponsibility, manipulation, suspiciousness, and unusual behavior and experiences) sometimes emerge as associated with the BPD syndrome. Such findings have led investigators to suggest that the AMPD trait algorithm for BPD might consider additional defining traits [26, 27]. However, our results are similar to that of Morey and Skodol [48], who found that the presence of four DSM-5 Section III traits increased convergence between DSM-IV-TR and DSM-5 Section III.

A recent meta-analysis conducted by Waters, Bagby and Sellbom [21] found that, of the seven Criterion B traits generating an AMPD diagnosis of BPD, six demonstrated significant associations with BPD. However, 11 of the 18 traits that are not now considered defining of BPD also showed significant associations with BPD. These findings are important and suggest refinement of the AMPD BPD algorithm may be in order, yet it is also relevant to note that the majority of the studies in Waters et al. [21] utilized a self-report instrument to assess the 25 pathological trait facets, rather than clinical ratings. Weighing evidence for revised AMPD diagnostic algorithms for BPD must also consider the method of ascertainment used in the source investigations. Self-report and clinical ratings for AMPD dimensions are not isomorphic. It is known that low to moderate correlations are found for self-report measures and informant report in PD with regard to externalizing disorders [49]. Our study relied on clinician ratings, using the full AMPD to characterize individual BPD criteria, and not whole person prototypes of BPD (or of actual patients independently diagnosed with BPD). Thus, the present study differs quite a bit from many of the studies in the aforementioned meta-analysis. Essentially, our results speak to how the BPD criteria are viewed in the language of the AMPD. This is a beginning consideration in the process of reckoning potential fine-tuning of AMPD schemes for BPD.

We found that the AMPD ratings of the BPD criteria demonstrate substantial intercorrelations. One way to summarize these relationships is through a PCA of the AMPD ratings of BPD criteria. Our PCA revealed two correlated components: a large first component characterized by dyscontrol and acting out (BPD criteria 2, 5, and 8), and another component defined by self-identity disturbance (BPD criteria 3, 7, and 9). Although our results are not directly comparable to those from studies using clinical ratings of patients, it is interesting that our results are so similar to those from previous factor analytic investigations of BPD criteria based on clinical data. For example, the confirmatory factor analytic (CFA) studies of Sanislow et al. [50] found three very highly correlated factors (labeled disturbed relatedness, behavioral dysregulation, and affective dysregulation), but a large central factor predominated. Our PCA of the AMPD-rated BPD criteria seems to capture variance related to dyscontrol and acting out (like the behavioral and affective dysregulation dimensions of Sanislow et al. [50]), as well as variance related to identity disturbance (like the disturbed relatedness dimension of Sanislow et al. [50]). Nonetheless, we emphasize that there was substantial shared variance within our AMPD-characterized BPD criteria (the first component carried 60% of the variance). This result resembles common findings from empirical factor analytic studies of BPD and PD criteria, which typically find a large general dimension [30, 50–54]. These studies varied widely as to samples and methods, but they consistently support the conclusion that a large general factor is present, and from one to two additional smaller factors may be reflected in the BPD criteria. Our study shows that when clinicians characterize the BPD criteria with the Criterion A and B of the AMPD, generally similar patterns emerge.

Our study reveals additional important details pertinent to the DSM criterion approach to diagnosis of BPD. Although the criteria for DSM-IV BPD diagnosis can yield a positive diagnosis in many ways (i.e., 256), we found that BPD AMPD profiles generated by (1) the grand mean of all nine criteria, (2) increasing numbers of criteria determined by base rate, (3) causal centrality, and (4) LPFS severity ratings of the BPD criteria resulted in highly similar AMPD profiles. We interpret this to mean that a substantial common core of AMPD-related variance acts as a virtual penumbra within the DSM BPD criteria, akin to a first factor or a general factor of pathology (p) within BPD [54]. It is important to note, however, that abstract ratings of individual BPD criteria may not capture the likely “severity” aspect that could be present when a patient with numerous positive BPD criteria is diagnosed with the AMPD scheme. For example, an individual with eight or nine positive BPD criteria likely shows greater psychopathology than the person meeting five diagnostic criteria [55]. Moreover, different combinations of criteria might be more “severe” than others (e.g., impulsivity and recurrent self-harm). Our study asked clinicians to evaluate the individual BPD criterion, and then averaged different configurations of individually rated BPD criteria in order to study potential differential AMPD relationships for BPD. In effect, we found that these procedures largely recreated the shared variance among the AMPD-BPD criteria, rather than flagging PD severity indicators such as higher LPFS or trait values as might be seen in a person evidencing numerous diagnostic criteria for BPD.

The current study is not without limitations. First, we asked participants to rate criteria of BPD only and not criteria for additional DSM PDs. Thus, we could not examine the BPD ratings in relation to other PD criteria ratings. Second, although not a limitation per se, our study specifically examined the interrelations between abstract BPD criteria, defined by AMPD ratings, and not clinical diagnoses of a whole person. Our main results precisely pertain to a criterion-based diagnosis and not to prototype-based diagnostic conceptions. In this regard, it is known that clinicians do not algorithmically apply DSM criteria in reaching diagnoses, and that the criteria are prone to be differentially weighted and applied [29, 56, 57]. Hence, additional studies of the AMPD with respect to BPD in naturalistic settings are needed.

Despite our more abstract focus on BPD criteria in this project, the results suggest generalizations that may be extended to diagnosis of BPD in clinical contexts. For example, the finding that various AMPD configurations of and different numbers of positive BPD criteria produced highly similar AMPD profiles is relevant. From this, we infer that in the clinical setting patients meeting threshold BPD diagnoses, but through different numbers of BPD criteria, likely will show similar AMPD profiles, with differences evident mainly in elevation on the key AMPD traits of anxiousness, depressivity, emotional lability, and impulsivity, along with elevations on LPFS domains. In other words, we suspect that the AMPD differences that may occur from increasing numbers of BPD criteria largely will reflect the elevation parameter and not the shape of the AMPD profile.

Additionally, we observed that raters did not make much use of the Delphi option. However, if a multi-tiered and -stepped Delphi procedure was followed, it is possible the group might have showed more change from their original ratings. A larger sample size of raters representing the population of general mental health practitioners might have made our results more generalizable to routine clinical practice settings. Yet, we note that our raters brought substantial experience and expertise to the task, arguably more so than represented in large-scale surveys using journeymen practitioners. Our results thus may speak more to expert application of the AMPD. We also note that a few of our raters seemed to have under emphasized the assumption that a hypothetical person demonstrating the BPD criteria (the actual targets for rating) possessed a PD-threshold LPFS score of 2. Nonetheless, our analyses showed that 95% of the raters rated the LPFS positive for five or more BPD criteria, suggesting our data reflect PD-level psychopathology.

Relatedly, we note that some AMPD traits that are generally associated with BPD (e.g., separation insecurity) did not reach significance in our study. We speculate perhaps our raters missed or underestimated certain theoretically relevant traits in the highly specific and abstract task of evaluating individual BPD criteria per se. As well, it may be the case that some such constructs in the nomological net of BPD, like abandonment fears and separation insecurity, require representation in more than one or two of the traditional DSM BPD criteria (see Gunderson [3] for suggestions on how to re-shape the traditional DSM criteria for BPD and achieve greater alignment with contemporary empirical findings; see also Gunderson, Herpertz, Skodol, Torgersen, & Zanarini [58].

On a more theoretical level, some have suggested [16, 17] that the AMPD may not be sufficiently comprehensive or theoretically differentiated to reflect the presumed nuanced nature of PDs. In fact, we agree this is an important line of investigation for future study and optimal levels of “granularity” for elements of the AMPD remain to be determined. We venture the suggestion that the preferred “granularity” of diagnostic elements will vary for the different constituencies of the scientist and clinician, and whose interests may include different investigative or clinical purposes. There are choice points in what, where, and how to locate diagnostic dimensions or criteria in terms of the multivariate space of PD psychopathology, when considered from the point of view of quantitative psychopathology [4]. As noted, different stakeholders and different purposes may inform different choice points. Be that as it may, our focus is more limited: We examined the cross-walking of the AMPD with traditional BPD diagnosis using the traditional definitional DSM criteria, rather than ways to refine or enlarge the scope of the AMPD.

## Conclusions

In sum, results of the current study demonstrate that the traditional DSM BPD criteria are capable of being translated into the AMPD metric and that substantial cross-connections occur for categorical and dimensional diagnostic nosological schemes for BPD. We suggest viewing the AMPD as a “new key” provides a way to bridge categorical and dimensional conceptualizations of BPD. This cross-walking of PD schemes reveals points of interdigitation, despite their origins in different models and traditions. The large general factor within BPD criteria [54] implies there is common ground between nosological conceptions of BPD, and the diagnosis generally is inferable from either scheme (see also Bastiaansen et al. [59]; Evans & Simms [20]). Importantly, remembering this commonality may contribute to improved communication between researchers and clinicians who may tend to favor one approach or another. This calls to mind Chang’s [60] philosophical logic that progress can occur when different, even competing, scientific paradigms are examined in parallel tracks. Whereas cogent rationales have been articulated by proponents of both PD nosological schemes [3, 5, 7], and the dimensional paradigm appears poised for growing acceptance in the field [4], our findings highlight the common ground between these points of view, at least for the diagnosis of BPD.

It will be interesting to see how empirical research and clinical application of the ICD-11 hybrid categorical-dimensional diagnosis for BPD plays out over time. The proposed BPD scheme utilizes clinical ratings for PD dimensions but permits a criterion-based and categorical Borderline Pattern to be appended for diagnoses of BPD. Our results suggest there may be substantial empirical redundancy between the trait ratings and the categorical specifier, but the latter may offer a degree of nosologically continuity as well as (important) cognitive ease or satisfaction for the practicing clinician [7], thus conferring clinical utility for the present time as diagnostic paradigms begin to shift [5].

## Data Availability

Data and relevant materials are available from the corresponding author upon request.

## Footnotes

1 We gratefully acknowledge the help and expertise of Robert F. Krueger, Ph.D., of the University of Minnesota.

2 We thank Donna S. Bender, Nicole M. Cain, Jenny Macfie, Robert M. Gordon, Jan H. Kamphuis, Mark F. Lenzenweger, John H. Porcerelli, Mark H. Waugh, and Aidan G. C. Wright for providing AMPD trait ratings of a prototypical patient with BPD (see [25]).

3 The 9 criteria can be combined 126 ways for 5 criteria, 84 ways for 6 criteria, 36 for 7 criteria, 9 for 8 criteria, and 1 for 9 criteria; this is a total of 256 possible combinations of criteria positive for BPD.

4 In the Grilo et al. [28] data, criterion 4 and 8 are tied for second highest BR, and 3 and 9 are tied for fifth place BR. Thus, the first five BPD criteria can be calculated straightforwardly, but the first “six” criteria include the ties for criteria 3 and 9, functionally becoming a total of seven criteria. Hence, we show the AMPD profiles based on five, seven, eight, and nine BPD criteria. We recognize that other empirical studies have found other BR frequencies of criteria [20, 30].

5 AMPD mean ratings of each individual BPD criterion are available from the first author upon request.

6 Relaxing the eigenvalue > 1 criterion permitted a 3-component solution to emerge (3rd eigenvalue = .96) accounting for 84% of the variance. This solution, with an oblique rotation, yielded components reflecting affective and interpersonal dyscontrol (criteria 1, 2, 8), identity disturbance (3, 7, 9), and behavioral dyscontrol (4,5). These results resemble the 3 highly correlated factors sometimes found in CFA from other studies (see Discussion section). We emphasize the 2-component interpretation of the PCA in the interests of descriptive parsimony. Moreover, the data were also studied with different rotations as well as a Principal Axes FA; results were generally quite similar.

7 The (divergent) Morey et al. [27] non-BPD AMPD profiles were for antisocial, avoidant, narcissistic, obsessive-compulsive, schizotypal, paranoid, schizoid PD (mean r = −.11; range .16 to −.45). The (divergent) Waters et al. [21] non-BPD AMPD profiles were for antisocial, avoidant, narcissistic, obsessive-compulsive, and schizotypal PD (mean r = .06; range .19 to −.01).

8 The rank order of the 1–9 DSM BPD criteria by BR from Grilo et al. [28] is: 6, 4 & 8 (tie), 7,2,3, 9, I, 5; the Kim and Ahn [29] causal centrality ranking order is: 4,5,1,8,9,2,6,3,7. The rank order of the BPD criteria by total LPFS AMPD severity ratings from our study is: 2 (tie), 4, 2 (tie), 8, 5,7,6.3,1. Spearman rho correlations are: BR and causal centrality = −.31, BR and total LPFS = .59, causal centrality and total LPFS = .26.

9 Note: DSM-IV and DSM-5, Section II use the same criteria for BPD.
